# The extended latissimus dorsi flap option in autologous breast reconstruction: A report of 14 cases and review of the literature

**DOI:** 10.4103/0970-0358.41107

**Published:** 2008

**Authors:** Mohammed A. Rifaat, Ayman A. Amin, Mahmoud Bassiouny, Ayman Nabawi, Sherif Monib

**Affiliations:** Department of Surgery, King Fahd Specialist Hospital, Dammam, Saudi Arabia; 1 National Cancer Institute, Cairo University, Fom el Khalig, Cairo, Egypt; 3 Alexandria University, Raml station, Alexandria, Egypt

**Keywords:** Breast reconstruction, extended latissimus dorsi flap

## Abstract

**Background:**

Autologous breast reconstruction using the extended latissimus dorsi flap has been infrequently reported. In the current study, the authors are reporting their own clinical experience with this method. A review of the literature is also discussed.

**Materials and Methods:**

Over a three year period, 14 patients underwent breast reconstruction using the extended latissimus dorsi (LD) flap. Patients with small to medium sized breasts were selected. The age of the patients ranged from 29 to 42 years with a follow-up period ranging from six to 18 months. The indications, flap-related complications and donor site morbidity and aesthetic results were evaluated.

**Results:**

The main indication to use the flap was dorsal donor site preference by patients. The remaining patients were either not suitable for a flap from the abdomen or wished to get pregnant and were offered the dorsal donor site. Neither total nor partial flap loss was recorded but donor site morbidity was mainly due to seroma, which was treated conservatively in all patients, except for one who required surgery. Another two patients suffered from wound breakdown and distal necrosis of the back flaps. Mild contour deformity was also noted on the back of all patients but caused no major concern. Indeed, the overall patient satisfaction was very high.

**Conclusion:**

The extended LD flap proved to be a good option for autologous breast reconstruction in selected patients. Patients should be warned of the potential for seroma and mild contour back deformity.

## INTRODUCTION

Breast reconstruction after mastectomy has been considered a very important step in the rehabilitation of breast cancer patients playing a major role in the interdisciplinary treatment for the disease.[1]

Various methods have been described in literature. They basically include tissue expanders, permanent implants in addition to various forms of autologous breast reconstruction. Autologous forms of reconstruction are recently gaining considerable interest in this patient group.[2]

The pedicled TRAM (transverse rectus abdominis muscle) flap is the preferred method for autologous breast reconstruction by many surgeons, particularly in the USA.[3] Indeed, better aesthetic results have been obtained by the free microvascular TRAM flap and more recently, the DIEP perforator flap (deep inferior epigastric artery flap) making them the gold standard in autologous breast reconstruction.[4–6]

The extended LD (Latissimus dorsi) flap is another option in autologous breast reconstruction. However, it has been infrequently reported and relegated to a second option in breast reconstruction in view of the excellent results and the great success in the last two decades of the other methods mentioned above.[7]

Nevertheless, pedicled and free TRAM or DIEP flaps may be contraindicated or not preferred by some patients. Indeed, the complex performance of microvascular procedures may not yet be possible in all centers.[7,8]

The current study was conducted to report our initial clinical experience with the extended LD flap in breast reconstruction with a better assessment for its indications, limitations, aesthetic outcome and donor site morbidity. A review of the literature is also discussed.

## MATERIALS AND METHODS

**Figure 1:**
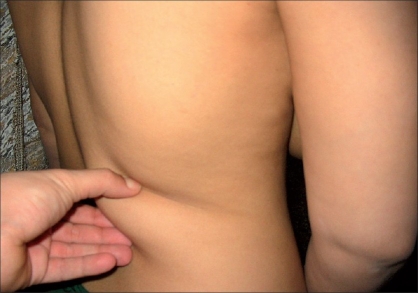
Skin pinch test to determine the thickness of adipose tissue on the back

From May 2003 to May 2005, 14 patients underwent breast reconstruction using the extended Latissimus Dorsi (LD) flap. Twelve patients were operated upon at the National Cancer Institute, Cairo University and two patients at King Fahd specialist Hospital in Dammam, Saudi Arabia by the principal author. Immediate reconstruction was done for twelve patients and two patients underwent delayed reconstruction. The age of the patients ranged from 29 to 42 years with a follow-up period ranging from six to 18 months [Table 1]. In addition to their history, physical examination, oncological assessment and fitness for operation, preoperative assessment included assessing the patients’ wish for various breast reconstruction options, state of local tissues, condition of the other breast and the state of the abdominal skin. Assessment of the subcutaneous tissues of the back by a preoperative skin pinch test and also evaluation of the subcutaneous fat of the iliac region were done. Patients with 2 cms thickness of adipose tissue of the back and fairly thick amount of fat in the iliac region were offered the option of the extended LD flap [Figure 1].

**Table 1:** Patient population

Age range (years)	29-42
Follow-up period	6-18 months
History of cancer	13/14
Postoperative irradiation	5/14
Immediate reconstruction	12/14
Delayed reconstruction	2/14

Patients with large-sized breasts with severe ptosis were excluded from the study and only those with small to medium-sized breasts were selected.

All patients gave their informed consent for the procedure and were aware of the potential complications and the possibility of secondary procedures.

The aesthetic results were assessed independently by the patients and two surgeons. The assessment by surgeons was determined by evaluating the preoperative and postoperative photographs for breast shape and contour, definition of the inframammary fold and the anterior axillary line, the creation of inferior fullness, the degree of symmetry to the other breast and the quality of the scars. The patients’ aesthetic evaluation was based on their subjective satisfaction with the shape of the new breast, the degree of symmetry to the contralateral breast, its consistency and the quality of the scars. The aesthetic results have been ranked into three categories by the surgeons: good, satisfactory and fair and satisfaction of patients has been classified into three levels: deeply satisfied, satisfied and poorly satisfied. Postoperative complications and their management and secondary operations required were also recorded.

### Technique

The technique is described elsewhere.[7–13] In immediate reconstruction, the thoracodorsal vessels are kept intact during axillary dissection whereas in delayed reconstruction, the integrity of the thoracodorsal vascular system should be checked from past surgical records. Indeed, a well-functioning Latissimus Dorsi muscle as determined by preoperative clinical examination is usually suggestive of an intact thoracodorsal neurovascular bundle.

**Figure 2:**
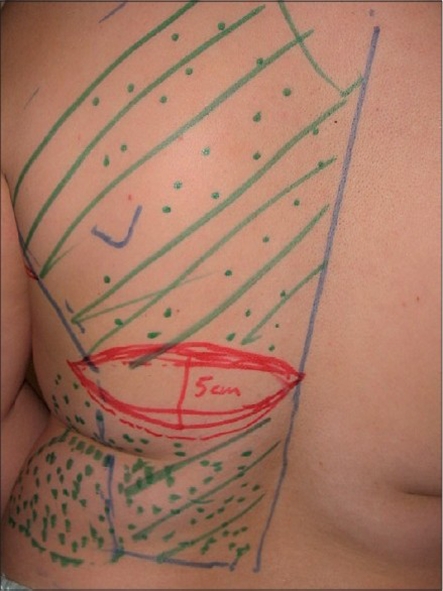
Preoperative markings while the patient is standing. Note the transversely oriented skin paddle and the dotted areas representing the adipofascial extensions of the scapular and the lumbar region

Various skin incisions and designs of skin islands have been described.[7–13] We used the transversely oriented skin paddle for our patients Preoperatively, the bra strap area and the inframammary crease were marked out with the patient standing. The transverse skin paddle was marked on the back by the pinch technique along the desired line [Figure 2]. The largest possible area of skin that would comfortably allow direct wound closure was marked out. The skin island was situated in the middle part of the muscle.

**Figure 3:**
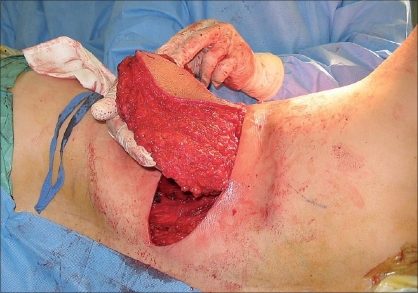
The muscle with the overlying fat after being harvested and transferred to the chest wall. As much fat as possible should be harvested

The flap was raised with the patient in the lateral decubitus position with a 90° abducted shoulder. The incision went down to the subdermal layer. The plane of dissection then continued along the subcutaneous plane just above Scarpa's fascia leaving at least one cm-thick native skin flaps. As much fat as possible should be harvested from the scapular region and the iliac region and the largest possible flap in terms of volume should be harvested with a tendency towards overcorrection [Figure 3].

The muscle was divided as usual from its attachments into the iliac crest and the thoracolumbar fascia. Its anterior border was then separated carefully from the underlying Serratus anterior muscle. The insertion of the muscle into the intertubercle groove on the humerus was subtotally divided to keep the pedicle protected by some fibers of the muscle and at the same time, minimize the axillary bulk which would be caused if whole insertion were to be left. This technique also allows for more reach of the muscle. The thoracodorsal nerve was preserved to minimize future atrophy of the muscle. The muscle with the overlying fat was now separated from all its attachments except at the intertubercle groove insertion and then mobilized to the chest wall through a subcutaneous tunnel, wide enough to introduce four fingers to reach to the site of reconstruction. Care was also taken not to disturb the inframammary fold in case immediate reconstruction was to be done.

The myoadipofascial flap was folded under the skin paddle in a way to provide the best possible projection with fullness mainly formed inferiorly to match the other breast. The muscle was anchored to the underlying muscle bed and the lateral contour of the breast mound was defined with the addition of some sutures to the lateral chest wall.

The back was closed in two layers over a large suction drain, which was usually left for two weeks postoperatively. Another suction drain was inserted under the transposed flap.

The wound of the chest wall was closed in layers and the flap was supported on the breast with some tapes laterally and superiorly.

## RESULTS

**Figure 4:**
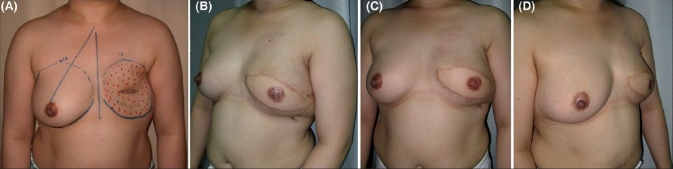
(A) Preoperative view of a patient presenting for delayed breast reconstruction with markings on the chest. (B-D) 12 months postoperative left oblique, front and right oblique views respectively

**Figure 5:**
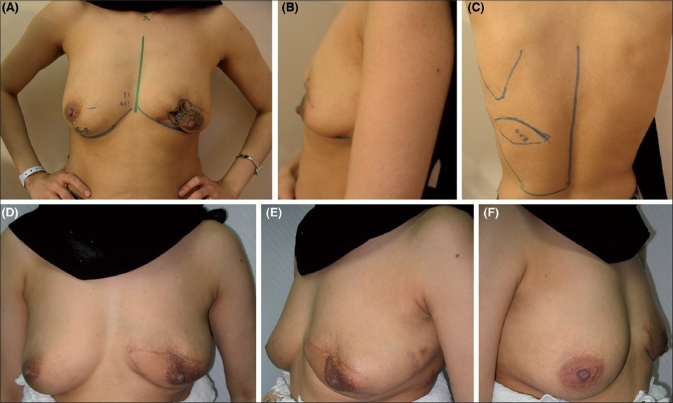
(A) Preoperative view of a patient presenting with recurrent left phllyoides tumor. Note marked contour deficiency due previous excisions. (B) Left lateral preoperative view. (C) Markings on the back. (D-F) Front, left oblique and right oblique views 4 months postoperatively after immediate

Immediate reconstruction was done for all patients except for two who underwent delayed reconstruction after modified radical mastectomy [Figure 4]. All patients suffered from breast cancer except one patient who had presented with a recurrent phylloides tumor of the breast for which she underwent partial mastectomy and immediate reconstruction [Figure 5]. Table 1 summarizes the patient population.

With regard to the reasons for choosing the extended LD flap; eight out of 14 patients preferred the dorsal donor site. Two other patients were thin for a TRAM flap and one had a significant abdominal scar that precluded a TRAM flap.

The remaining three patients wished to become pregnant in the future and neither wished to be subjected to any possible donor site morbidity from a pedicled TRAM flap with the potential for abdominal wall weakness nor to undergo a complex free tissue transfer and hence have preferred to have the LD flap option.

**Figure 6:**
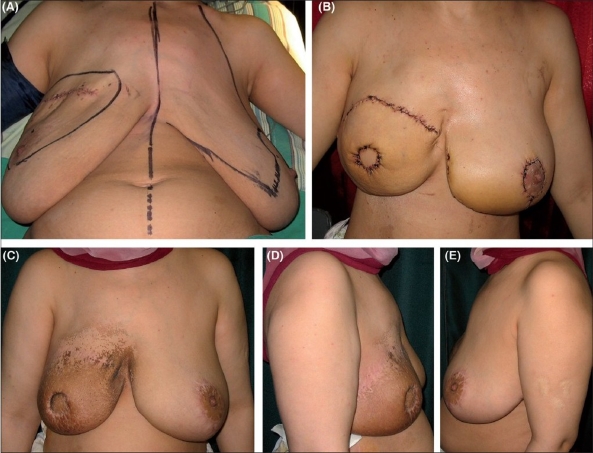
(A) Preoperative view of a patient with right breast cancer diagnosed by previous lumpectomy. (B) 3 weeks postoperative view after right skin sparing mastectomy and immediate reconstruction, with contralateral simultaneous inferior pedicle breast reduction. (C-E) Front, right lateral and left lateral views six months postoperatively. Note postirradiation effects on the flap

In all cases, the skin island of the LD flap had reconstructed a part of the skin of the breast. In only one case with a large skin envelope, it was possible to achieve subtotal burying of the flap keeping a disc of the cutaneous paddle of the flap, which corresponded to the future areola [Figure 6].

### Flap-related complications

There was no total or partial flap loss in this study and fat necrosis was noted in two patients after three months [Table 2]. This was clinically palpable during routine clinical follow up as a firm mass in the superficial part of the buried LD flap under the native chest wall or breast flaps. Malignancy was then excluded by FNAC.

**Table 2:** Complications

*Complication*	*Incidence*	*Surgery required*
*Flap-related*		
Fat necrosis	2/14	None
*Donor site-related*		
Seroma	9/14	1/14
Distal necrosis of black flaps	2/14	1/14

### Donor site morbidity

The most common donor site problem was seroma, which occurred in nine cases. All patients were treated conservatively by repeated aspiration in the clinic except for one patient, who required surgery by curettage of the cavity wall formed by the chronic seroma. Another two patients suffered from wound breakdown and edge necrosis of the back flaps [Table 2]. One of them required reoperation while the wound of the other patient healed spontaneously after six weeks following conservative eschar separation and local wound care. Most patients had temporary limitation of shoulder movements postoperatively but all recovered completely within three weeks.

In the current study, nearly all patients showed a minor residual contour deformity in the back as a result of fat harvesting at the flap’s site. This was mainly noticeable in the iliac region. It improved with time when the tissues got more lax and less stretched. Furthermore, with some later effects of gravity on the superior skin folds the deformity became less noticeable. Nevertheless, it was never a cause of any concern to the patients.

### Ancillary procedures

The majority of patients underwent no surgical intervention for the opposite breast except one patient who presented with bilateral severe ptosis and a moderate breast size requiring simultaneous contralateral breast reduction at the time of mastectomy and reconstruction to avoid noticeable asymmetry. At the same time, her large breast skin envelope following skin-sparing mastectomy was utilized to allow for subtotal burying of the flap [Figure 6]. Another patient was offered contralateral surgery due to asymmetry because of size mismatch and moderate ptosis but she refused.

Two patients underwent secondary nipple and areola reconstruction. One with a local skate flap and tattooing and the other with local dermal flaps and full-thickness skin graft from the groin crease [Figure 4].

### Aesthetic outcome

The aesthetic evaluations by the patients and the surgeons are summarized in Table 3. Those who had some mild to moderate postoperative asymmetry in the sizes of both breasts and those who received postoperative radiation on the flap with consequent edema of the skin and firmness of the flap were the ones who had suboptimal aesthetic results. The results were graded as being slightly less favorable by the surgeons but the overall satisfaction by patients was fairly high. Nipple and areola reconstruction in two patients increased aesthetic outcome and patient satisfaction.

**Table 3:** Evaluation of aesthetic results by patients and surgeons

*Scoring by patients and surgeons*	*Number of patients*
Patients	
Deeply satisfied	9/14
Satisfied	4/14
Poorly satisfied	1/14
Surgeons	
Good	6/14
Satisfactory	5/14
Fair	3/14

## DISCUSSION

Breast reconstruction with autogenous tissues is known to provide a much more natural, durable and long-lasting option for patients.[2]

The latissimus dorsi (LD) flap was first described in the seventies for breast reconstruction.[14,15] It has since become a common practice to increase the volume of the standard LD by the addition of a breast implant to compensate for the small volume provided by the classical flap.

Although the technique is quick and easy with an aesthetically pleasing outcome, negative sequelae associated with breast implants such as capsular contracture, implant displacement and rupture can still potentially occur.[16] The rate of capsular contracture has been variably reported in the literature and ranges from 20 to 40% in some studies.[17,18] To avoid the addition of an implant to the LD flap, attempts have been made to increase the volume of the flap with autogenous tissues.

Early attempts to increase the volume of the flap by including fascial extensions were described by Hokin in 1983 and then by Hokin and Sliverskiold in 1987 taking the whole muscle and lumbar fascia with the largest possible skin paddle running obliquely along the back. The skin paddle could be partly or wholly de-epithelialized for added volume.[19,20]

In 1984, Marshall *et al*. described a latissimus dorsi flap with a Y-shaped skin paddle with the vertical end of the Y being de-epithelialised and turned below the upper skin paddle to provide added projection.[9] In 1988, Papp *et al*. took the entire muscle with the largest possible horizontal skin paddle, which was also de-epithelialised, turned inward and covered with the Latissimus muscle.[10]

McCraw and Papp then described a series of Latissimus dorsi myocutaneous flaps with different skin paddle designs. The skin paddle can be a horizontal, crescentic or fleur-de-lis-shaped skin paddle. The fleur-de-lis-shaped design was also partly de-epithelialised to add to the volume provided by the muscle, the iliac crest fat and the fat over the Trapezius muscle.[21,22]

Further emphasis on the role of the parascapular and scapular fat “fat fascia” was then shown by Germann *et al*. and Heitmann *et al*. The authors stressed the superiority of the parascapular and scapular “fat fascia” to the lumbar fat with regard to the blood supply, which is random in the latter. The skin paddle was designed either as a fleur-de-lis or horizontal skin patterns.[8,23]

In the current study, we have designed the skin paddle in a transverse direction and we were still able to harvest enough fat from the scapular and lumbar regions. The transverse scar was quite acceptable to patients.

It is to be noted that the choice of the skin design varies from one surgeon to another. Some authors have abandoned the use of the fleur-de-lis skin paddle design because of the resulting extensive donor-site scar and have adopted to use the transverse skin paddle instead, where it can be hidden in the bra line.[7,11,24] Others have preferred the crescent-shaped paddle described by Marshall *et al*.[9,12]

We have dissected the dorsal skin flaps above the Scarpa’s fascia with at least one cm thickness of the retained dorsal skin flaps. Some authors mentioned that one to two mm back flap thickness over a five cm radius is sufficient to preserve the vascularity of the flaps.[11,21] We agree with McGraw, Chang and their co-workers that one cm-thick dorsal skin flaps should be left behind.[7–21]

Nevertheless, dorsal flap necorsis is a potential problem and it has been variably reported by several authors. Chang *et al*. reported 16% necrosis rates in 75 patients while Delay *et al*. reported 3% incidence in 100 patients.[7,12] In the current report, two cases developed necrosis of the edges of the flaps in the back. One of them required reoperation while the wound of the other patient healed spontaneously. It is important that the primary wound closure of the donor site should be relatively tension-free.[7,11–12] The optimum width of the skin paddle is hard to estimate in terms of numbers but this varies from one patient to the other and it usually lies in the range of seven to nine cm.[12]

We believe that inadvertently excessive thinning of back flaps as well as greater tension created in wound closure due to poor skin paddle design have resulted in necrosis and wound breakdown in the latter two cases.

On the other hand, the LD flap itself is a very reliable flap with very low incidence of partial or complete necrosis.[11,25] One large study quoted complete loss of the latissimus flap in one of 125 patients[22] while another study reported 1% total flap loss in a series of 100 patients.[12] Although the flap can rely on reverse flow from the serratus anterior branch, the extended LD flap with the large additional cuff of fat requires extra circulation to ensure full viability. Hence, the integrity of the thoracodorsal pedicle should always be sought.[11,21] In case of doubt, particularly in the context of delayed reconstruction, some authors have recommended angiography and ultrasonography of the vascular pedicle.[8,11] This was not necessary in the two cases that underwent delayed reconstruction in this study as the integrity was established clinically and confirmed by primary operative records.

Neither complete nor partial necrosis occurred in any case in this report. Necrosis usually happens when there is tension or twist on the pedicle. Some believe that keeping the humeral tendon insertion attached can minimize the occurrence of this problem. Indeed, the extended LD flap is bulkier with the additional fat and it requires less mobilization making the division of the humeral insertion unnecessary in most cases. The division should be considered only in few cases when it is necessary to obtain adequate excursion of the extended latissimus dorsi flap or in thin patients where the bulkiness in the axilla may be readily noticeable. Furthermore, the bulkiness of the axilla created by the undivided tendon will decrease with time as the muscle atrophies.[7] The latter authors have chosen to divide the nerve in their series to prevent potential postoperative involuntary muscle contraction and hence they have expected future considerable decrease of the bulkiness in the axilla.

Nonetheless, many surgeons have advocated division of the humeral insertion of the Latissimus dorsi tendon and all the branches of the thoracodorsal vessels in an attempt to improve the excursion and rotation of the flap.[8,20,21,24,26] This is also believed to eliminate the “bulky” stump in the axilla which is sometimes described by some as holding a book under the arm. Once the flap is fixed to the chest wall, tension on the pedicle should be eliminated.[8,26]

We agree with Delay *et al*. that subtotal division of the LD tendon leaving a small muscle bridge to protect the pedicle would be a reasonable alternative. This may help to minimize the bulk while still offering some protection to the pedicle. This technique should lead to only a temporary bulge which improves considerably with time as it is expected that a degree of muscle disuse atrophy will occur to some extent despite keeping the thoracodorsal nerve intact.[12]

It is noteworthy that a higher incidence of fat necrosis is expected in larger flaps due to the harvest of some fat from beyond the borders of the muscle with its random blood supply.[7] The reported incidence however, is generally lower than that of the TRAM flap which can reach 10.6%.[27] Delay *et al*. reported 4% incidence of fat necrosis in their series of 100 patients while Menke *et al*. reported only 2% in 125 patients.[12,24]

Two patients out of 14 developed fat necrosis in this study. The diagnosis was a clinical one. The lesion was discovered during routine regular follow up examination as a palpable firm mass in the substance of the flap under the native breast or chest wall skin flaps. This was confirmed by **FNAC**. Despite the smaller sample size of this study, the incidence of fat necrosis is considered relatively high. Perhaps this could be attributed to the high index of suspicion by our surgical oncology colleagues during clinical follow-up.

The need for overcorrection at the time of reconstruction should not be overlooked as the flap decreases in volume in late follow-up by about 20 to 25%, reaching the least value in 12 months.[8] This is thought to be due to muscle atrophy and some authors believe that preservation of the thoracodorsal nerve may help to preserve part of the muscle bulk.[8,12,22] Others argue against preservation of the nerve as occasional twitches of the muscle that may occur later, may be troublesome to some patients.[7]

The proponents of nerve preservation believe that consequent muscle twitches will fade progressively over time and only rarely is secondary nerve transection required.[8,12,24]

We have preserved the nerve in all cases in this series aiming to minimize a substantial loss in the volume of the muscle due to its denervation. Only one patient was noted to complain of these involuntary muscle twitches but this also improved spontaneously with time.

In so far as the vascularity is concerned, the LD flap tolerates the postoperative irradiation well.[13] However, this certainly has a negative effect on the cosmetic outcome. Postirradiation fibrosis, soft tissue necrosis, edema and contractures can all develop and alter the shape and the consistency of various flaps after breast reconstruction. Unfortunately, not all patients choosing immediate reconstruction after mastectomy are known preoperatively to be going for postoperative radiation therapy.[28]

The overall patient satisfaction in this study was very good. Nine patients were deeply satisfied and four were satisfied. The only poorly satisfied patient in this study suffered distal necrosis of the dorsal flaps with consequent wound breakdown, which necessitated surgical intervention. Also, she had a suboptimal aesthetic result because of the asymmetry between both breasts due to underestimation of the contralateral breast ptosis in addition to the postoperative irradiation effect on the flap. The asymmetry could have been improved by contralateral breast surgery but the patient refused.

On the other hand, the results were graded as being slightly less favorable by the surgeons due to their more critical look searching for mild asymmetry or postoperative radiation morphologic changes on the flap. Although the tissue edema and fibrosis were more severe in the early postirradiation period, the reconstructed breasts got softer with time and most patients were satisfied.

The patients in this report who had mild to moderate asymmetry were very reluctant to undergo simultaneous or delayed contralateral breast surgery. This may also reflect the Middle Eastern culture of our patients who have a particular fear to undergo operation on the contralateral normal breast. Nonetheless, contralateral surgery was strongly indicated in two patients due to severe ptosis of the other breast. Only one patient agreed and was deeply satisfied despite obvious radiation morphologic changes [Figure 6].

Delay *et al*. reported that the majority of their patients did not require contralateral breast surgery.[12] This reflects one of the advantages of autogenous reconstruction, which is the creation of natural breast ptosis [Figure 4].

It is well noted that completion of nipple/areolar reconstruction improves patient aesthetic satisfaction with their breast reconstructions.[29] On the other hand, a large number of patients may just be satisfied by the newly constructed breast mound and may refuse the option of nipple and areola reconstruction.[30] Only two patients in this study were willing to undergo nipple and areola reconstruction. This certainly improved the cosmetic scoring by both patients and the surgeons.

The main indication of the flap in this study was patients’ preference in 57% of cases (8/14). Similarly Delay *et al*. reported dorsal donor site preference in more than half of their patients.[12] The prolonged recovery following the pedicled TRAM flap, the possibility of using a mesh and the chance of developing hernia have discouraged some of our patients from making it as their first choice.

Three of our patients in the childbearing age who were otherwise good candidates for a TRAM flap were very hesitant to have this choice for fear of any potential abdominal wall complication and preferred the extended LD flap option.

Although there is no substantial evidence to show any untoward effects of pregnancy on reconstruction, there are only few reports of patients having normal full term pregnancies and deliveries following pedicle or free transverse rectus abdominis myocutaneous (TRAM) flap breast reconstruction and the literature is limited in this regard as pregnancy after breast cancer seems relatively uncommon.[31–32]

Indeed, many patients have refused the free flap choice because of the complex nature of the procedure.

As it is expected that the extended LD flap provides more volume than the standard LD flap, its use is warranted in partial mastectomy or breast contour defects following previous unsatisfactory reconstructions by other methods.[14]

In this study, only one case underwent immediate reconstruction after partial mastectomy with a satisfactory outcome [Figure 5].

The disadvantages of the extended LD flap lie in its donor site morbidity. Noticeably, this includes a high incidence of seroma in comparison to the standard LD flap ranging from 9 to 19% in some studies and up to 79% in others.[2,8,12] Indeed, the rate was noted to be higher in obese patients.[12]

The incidence of seroma in this report reached 64% (9/14). This seroma though uncomfortable to patients, is easy to manage and may settle after a few aspirations.

Some have advocated stitching the elevated back flaps to the undersurface at multiple levels (quilting stitches); others believe that leaving the drain a bit longer (two weeks) may help to minimize the seroma.[11,33] In the current study, we routinely left the drain for nearly two weeks, but in spite of that, the incidence of seroma was still high.

Another potential problem following extended LD is the contour deficiency on one side of the back.[7,8,12] Although this is slight and usually settles when the back becomes supple and lax with time, it is sometimes more obvious especially in obese patients.[12]

In the current study, this deformity was noted on the back of most patients. However, it was minimal and acceptable. In terms of shoulder function, the functional deficit is usually low whether a standard or an extended LD flap is used.[12–34] The flap should however, be avoided in professional swimmers.[34]

In conclusion, the extended LD flap is another good alternative that can be offered for autologous breast reconstruction. The flap is primarily indicated for those who are not suitable candidates for TRAM flaps or for that group of patients who would prefer the back donor site and are reluctant to proceed for the prolonged recovery of the pedicled TRAM or for the possible morbidity and the complexity of free tissue transfers. The disadvantages of the flap lie in the high incidence of seroma which usually responds to repeated aspirations. Also noted is the mild contour deficiency of the back, which usually improves with time and may be acceptable to most patients. However, obese patients should be warned that this deformity may be more obvious on their backs. Other disadvantages are the limitations in the size of the flap making it unsuitable for certain groups of patients who have very large and/or severely ptotic breasts unless a contralateral procedure (reduction/mastopexy) is to be done.

## References

[1] Kurtz JM, Amalric K, Brandone H, Ayme Y, Jacquemier J, Pietra JC (1989). Local recurrence after breast-conserving surgery and radiotherapy: Frequency, time, course and prognosis. Cancer.

[2] Trabulsy PP, Anthony JP, Mathes SJ (1994). Changing trends in postmastectomy breast reconstruction: A 13-year experience. Plast Reconstr Surg.

[3] Beasley ME (1994). The pedicled TRAM as preference for immediate autogenous tissue breast reconstruction. Clin Plast Surg.

[4] Kroll SS, Schusterman MA, Reece GP, Miller MJ, Smith B (1994). Breast reconstruction with myocutaneous flaps in previously irradiated patients. Plast Reconstr Surg.

[5] Blondeel PN, Vanderstraeten GG, Monstrey SJ, Van Landuyt K, Tonnard P, Lysens R (1997). The donor site morbidity of free DIEP flaps and free TRAM flaps for breast reconstruction. Br J Plast Surg.

[6] Allen RJ, Spear SL (1998). Perforator flaps in breast reconstruction. Surgery of the breast: Principles and art.

[7] Chang DW, Youssef A, Cha S, Reece GP (2002). Autologous breast reconstruction with the extended latissimus dorsi flap. Plast Reconstr Surg.

[8] Germann G, Steinau HU (1996). Breast reconstruction with the extended latissimus dorsi flap. Plast Reconstr Surg.

[9] Marshall DR, Anstee EJ, Stapleton MJ (1984). Soft tissue reconstruction of the breast using an extended composite latissimus dorsi myocutaneous flap. Br J Plast Surg.

[10] Papp C, Zanon E, McCraw J (1988). Breast volume replacement using the deepithelialized latissimus dorsi myocutaneous flap. Eur J Plast Surg.

[11] Barnett GR, Gianoutsos MP (1996). The latissimus dorsi added fat flap for natural tissue breast reconstruction: Report of 15 Cases. Plast Reconstr Surg.

[12] Delay E, Gounot N, Bouillot A, Zlatoff P, Rivoire M (1998). Autologous latissimus breast reconstruction: A 3-year clinical experience with 100 patients. Plast Reconstr Surg.

[13] Aitken ME, Mustoe TA (2002). Why change a good thing? Revisiting the fleur-de-lis reconstruction of the breast. Plast Reconstr Surg.

[14] Olivari N (1976). The latissimus flap. Br J Plast Surg.

[15] Schneider WJ, Hill HL, Brown RG (1977). Latissimus dorsi myocutaneous flap for breast reconstruction. Br J Plast Surg.

[16] Smith BK, Cohen BE, Biggs TM, Suber J (2001). Simultaneous bilateral breast reconstruction using latissimus dorsi myocutaneous flaps: A retrospective review of an institutional experience. Plast Reconstr Surg.

[17] McCraw JB, Maxwell GP (1988). Early and late capsular “deformation” as a cause of unsatisfactory results in the latissimus dorsi breast reconstruction. Clin Plast Surg.

[18] DeMey A, Lejour M, Declety A, Meythiaz AM (1991). Late results and current indications of latissimus dorsi breast reconstructions. Br J Plast Surg.

[19] Hokin JA (1983). Mastectomy reconstruction without a prosthetic implant. Plast Reconstr Surg.

[20] Hokin JA, Silfverskiold KL (1987). Breast reconstruction without an implant: Results and complications using an extended latissimus dorsi flap. Plast Reconstr Surg.

[21] McCraw JB, Papp C, Edwards A, McMellin A (1994). The autogenous latissimus breast reconstruction. Clin Plast Surg.

[22] Papp C, McCraw JB (1998). Autogenous latissimus breast reconstruction. Clin Plast Surg.

[23] Heitmann C, Pelzer M, Kuentscher M, Menke H, Germann G (2003). The extended latissimus dorsi flap revisited. Plast Reconstr Surg.

[24] Menke H, Erkens M, Olbrisch RR (2001). Evolving concepts in breast reconstruction with latissimus dorsi flaps: Results and follow-up of 121 consecutive patients. Ann Plast Surg.

[25] Roy MK, Shrotia S, Holcombe C, Webster DJ, Hughes LE, Mansel RE (1998). Complications of latissimus dorsi myocutaneous flap breast reconstruction. Eur J Surg Oncol.

[26] Gerber B, Krause A, Reimer T, Müller H, Friese K (1999). Breast reconstruction with latissimus dorsi flap: i0 mproved aesthetic results after transection of its humeral insertion. Plast Reconstr Surg.

[27] Watterson PA, Bostwick J, Hester TR, Bried JT, Taylor GI (1995). TRAM flap anatomy correlated with a 10 year clinical experience with 556 patients. Plast Reconstr Surg.

[28] Kronowitz SJ, Robb GL (2004). Breast reconstruction with postmastectomy radiation therapy: Current issues. Plast Reconstr Surg.

[29] Shaikh-Naidu N, Preminger BA, Rogers K, Messina P, Gayle LB (2004). Determinants of aesthetic satisfaction following TRAM and implant breast reconstruction. Ann Plast Surg.

[30] Mathes SJ, Ueno CM, Mathes SJ, Hentz VR (2006). Reconstruction of the nipple-areola complex. Plastic Surgery.

[31] Johnson RM, Barney LM, King JC (2002). Vaginal delivery of monozygotic twins after bilateral pedicle TRAM breast reconstruction. Plast Reconstr Surg.

[32] Collin TW, Coady MS (2006). Is pregnancy contraindicated following free TRAM breast reconstruction?. J Plast Reconstr Aesthet Surg.

[33] Titley OG, Spyrou GE, Fatah MF (1997). Preventing seroma in the latissimus dorsi flap donor site. Br J Plast Surg.

[34] Russell RC, Pribaz J, Zook EG, Leighton WD, Eriksson E, Smith CJ (1986). Functional evaluation of latissimus dorsi donor site. Plast Reconstr Surg.

